# Experimental warming and its legacy effects on root dynamics following two hurricane disturbances in a wet tropical forest

**DOI:** 10.1111/gcb.15870

**Published:** 2021-09-26

**Authors:** Daniela Yaffar, Tana E. Wood, Sasha C. Reed, Benjamin L. Branoff, Molly A. Cavaleri, Richard J. Norby

**Affiliations:** ^1^ Ecology and Evolutionary Biology University of Tennessee Knoxville Tennessee USA; ^2^ Environmental Sciences Division and Climate Change Science Institute Oak Ridge National Laboratory Oak Ridge Tennessee USA; ^3^ USDA Forest Service International Institute of Tropical Forestry Río Piedras Puerto Rico; ^4^ Southwest Biological Science Center U.S. Geological Survey Moab Utah USA; ^5^ Gulf Ecosystem Measurement and Modeling Division Environment Protection Agency Gulf Breeze Florida USA; ^6^ College of Forest Resources and Environmental Science Michigan Technological University Houghton Michigan USA

**Keywords:** belowground, climate change, luquillo experimental forest, multiple disturbances, root traits, wet forest

## Abstract

Tropical forests are expected to experience unprecedented warming and increases in hurricane disturbances in the coming decades; yet, our understanding of how these productive systems, especially their belowground component, will respond to the combined effects of varied environmental changes remains empirically limited. Here we evaluated the responses of root dynamics (production, mortality, and biomass) to soil and understory warming (+4°C) and after two consecutive tropical hurricanes in our in situ warming experiment in a tropical forest of Puerto Rico: Tropical Responses to Altered Climate Experiment (TRACE). We collected minirhizotron images from three warmed plots and three control plots of 12 m^2^. Following Hurricanes Irma and María in September 2017, the infrared heater warming treatment was suspended for repairs, which allowed us to explore potential legacy effects of prior warming on forest recovery. We found that warming significantly reduced root production and root biomass over time. Following hurricane disturbance, both root biomass and production increased substantially across all plots; the root biomass increased 2.8‐fold in controls but only 1.6‐fold in previously warmed plots. This pattern held true for both herbaceous and woody roots, suggesting that the consistent antecedent warming conditions reduced root capacity to recover following hurricane disturbance. Root production and mortality were both related to soil ammonium nitrogen and microbial biomass nitrogen before and after the hurricanes. This experiment has provided an unprecedented look at the complex interactive effects of disturbance and climate change on the root component of a tropical forested ecosystem. A decrease in root production in a warmer world and slower root recovery after a major hurricane disturbance, as observed here, are likely to have longer‐term consequences for tropical forest responses to future global change.

## Graphical abstract

Our study measured root dynamics in response to a field experimental warming and two consecutive hurricanes, Irma and María, in a tropical forest of Puerto Rico. We found that root biomass and production decreased with experimental warming in the 7 months before the hurricanes. Following the hurricane disturbances, root biomass and production increased, but less in the previously warmed plots compared to control plots, suggesting a warming legacy effect on root recovery. These unprecedented results show the complex interactive effects of disturbance and climate change on the root component of a tropical forested ecosystem.

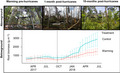

## 1 INTRODUCTION

In the anthropocene, ecosystem disturbances rarely occur in isolation, and the interactions between multiple disturbances can influence ecosystem functions in novel ways when compared to the effects of individual disturbances alone (Beard et al., 2005; Ratajczak et al., 2018). Climate change and hurricanes are prominent examples: human‐caused warming of the atmosphere and oceans is leading to increases in the intensity and frequency of hurricanes (Emanuel, 2013). Warming of terrestrial ecosystems is a perturbation likely to affect ecosystem function through changes in carbon and nutrient cycling, including belowground processes (Adachi et al., 2017; Nottingham et al., 2020), which can influence plant recovery after an additional disturbance (e.g., hurricane). Understanding the effects and mechanisms driving an ecosystem through multiple disturbances is a key to better predicting future scenarios of ecosystem change, as well as feedbacks to future climate.

Increased stress and depletion of energy reserves from exposure to elevated temperatures may be especially important in tropical ecosystems, which are soon expected to be subjected to temperatures never before experienced (Cavaleri et al., 2015). Tropical forests account for a disproportionate amount of global terrestrial primary production. Because of the large magnitude of carbon exchanges between tropical forests and the atmosphere (Beer et al., 2010; Foley et al., 2003), small relative changes in flux from these forests can have a considerable influence on atmospheric CO_2_ concentration (Cavaleri et al., 2015; Raich & Schlesinger, 1992; Wood et al., 2012), which is the principal driver of climate change. Furthermore, the carbon balance of some tropical ecosystems is already reported to have been affected by multiple climate disturbances including hurricanes (Harris & Lugo, 2012; Malhi et al., 2014). Recovery of forest carbon budgets and fluxes, including plant roots biomass, production, and turnover can take from 10 to 60 years following a major hurricane disturbance (Lugo et al., 2000; Parrotta & Lodge, 1991; Silver & Vogt, 1993; Teh et al., 2009). Yet, no in situ study in the tropics has considered the effects of warming on root dynamics, nor the effects of antecedent warming on root recovery from hurricane disturbance.

Increasing temperatures can affect root production directly or indirectly through alterations in carbon assimilation, metabolic processes, water input, and nutrient mineralization (Burton & Pregitzer, 2003; Fitter et al., 1999; Piatek & Allen, 2010; Pregitzer et al., 2000; Tierney et al., 2003; Zhou et al., 2011). The effects of warming, however, are not consistent across plant species or forest type. For example, some studies show that high temperature can increase root mortality rates due to higher evapotranspiration, leading to water shortage effects (Fitter et al., 1999; Forbes et al., 1997; Majdi & Öhrvik, 2004; Pritchard et al., 2000; Wan et al., 2004). Others show an increase in root production that is possibly due to enhanced nutrient availability or photosynthesis stimulation (Majdi & Öhrvik, 2004; Malhotra et al., 2020; Pugnaire et al., 2019; Wan et al., 2004; Wang et al., 2021; Zhou et al., 2012), or no response in root dynamics to soil warming (Dukes et al., 2005). However, most manipulative warming studies have focused on single‐factor experiments, mostly in temperate and boreal ecosystems, with highly variable results (Norby et al., 2004; Zhou et al., 2012). In fact, to the best of our knowledge, no studies in any biome have measured the consecutive effects of both warming and hurricane disturbance on belowground function.

Given the uncertainty surrounding climatic influences on root dynamics, we developed this study in an in situ warming experiment in a tropical forest (Puerto Rico), the Tropical Responses to Altered Climate Experiment (TRACE). The warming treatment was applied for 1 year and root images were collected for 7 months before Hurricanes Irma and María passed over the island in September 2017, which halted the warming treatment. The hurricane events provided an extraordinary opportunity to explore how previously warmed forest plots would recover following hurricane disturbance. We followed the root system response for 10 months following hurricane disturbance with no warming applied, which enabled us to evaluate potential legacy effects of prior warming on the recovery process.

The initial objectives of this study were to investigate how root biomass, root production, and root mortality responded to +4°C warming treatment. After the hurricanes, our additional objectives were to measure how these root dynamics responded to hurricane disturbances and to assess whether and how prior warming had resulted in legacy effects. Finally, we assessed how these responses changed with environmental variables (e.g., soil microclimate and soil nutrient status) that have the potential to affect root dynamics.

## 2 MATERIALS AND METHODS

### 2.1 Study site

The experimental site is located within the Luquillo Experimental Forest, in northeastern Puerto Rico (LEF; 18°19′48″N, 65°43′48″W). The area is considered “sub‐tropical” according to the life zones of Holdridge, and “tropical” according to the climate classification of Köppen‐Geiger, and Trewartha (Ewel & Whitmore, 1973; Jacob et al., 2012; Kottek et al., 2006). The site is in a secondary forest that had been recovering from deforestation for approximately 70 years at the time of this study, it is approximately 100 m above sea level, receives on average 3300 mm of rainfall annually, and has a mean annual temperature of 24°C, with little variation throughout the year (Garcia‐Martino et al., 1996; Kimball et al., 2018). The soils, derived from volcanoclastic sediments, are Ultisols and are clay‐rich and high in aluminum and iron (Brown et al., 1983; Scatena, 1989). Prior to Hurricanes Irma and María, common mature tree species in this forest were *Prestoea montana*, *Psychotria brachiata*, *Syzygium jambos*, and *Sloanea berteriana*, with an average diameter of 8 cm, a mean canopy height of 20 m (Cook et al., 2013), and canopy openness of ~10% (Reed et al., 2020). In Puerto Rico, >80% of fine root biomass is typically found in the first 20 cm of soil depth (Yaffar & Norby, 2020).

### 2.2 The warming experiment

The experiment has six plots, three control and three warmed, which are 12 m^2^ each (Kimball et al., 2018). The heated plots have a hexagonal array of six infrared (IR) heaters each (Model Raymax 1010, Watlow Electric Manufacturing Co.) at a height of approximately 3.6 m from the ground. Prior to the hurricanes, these heaters uniformly warmed the understory and soils up to 4°C above ambient temperatures. Plot locations were selected in the understory to avoid stems of mature canopy trees. However, the roots measured in the plots could originate from understory plants that are located inside the plots as well as both understory plants and mature trees located outside the plots. The warming treatment started in September of 2016 (Figure S1). Each plot has soil temperature and moisture sensors at three depths (0–10, 20–30, and 40–50 cm), in addition to air temperature and relative humidity sensors. Soil temperatures were on average 3.6°C warmer than controls at 0–10 cm depth, and ~3°C warmer than control at 20–50 cm in depths after 1 month of warming (Kimball et al., 2018). There was no significant differences in mean soil moisture between the control and heated plots in the first 10 cm, but heated plots were approximately 0.036 m^3^ m^−3^ drier than control plots at both 20–30 cm and 40–50 cm depths (Kimball et al., 2018). The design and detailed description of the plot installation can be found in Kimball et al. (2018). Prior to and during the initial year of warming, the TRACE team collected climate and soil microclimate data (Kimball et al., 2018), soil biogeochemistry data (Reed et al., 2020), aboveground plant demography and physiology data (Bachelot et al., 2020; Carter et al., 2020; Kennard et al., 2020), and belowground plant data, including root dynamics (present study).

We used three different methods to measure different root traits: minirhizotron tubes for root biomass and dynamics (root production, mortality, and turnover), soil cores for root biomass and root mass per length (RML), and root in‐growth cores for root morphology. In each plot, we installed two acrylic minirhizotron tubes (Pena‐Plas, Pittsburgh, Pennsylvania, USA) of 5.1 cm inner diameter and 1.8 m long with a water‐tight plug at the bottom, at 45° angle from the horizontal up to 1 m deep (as described in Norby et al., 2004). The installation took place in February 2015 (Figure S1), more than 1.5 years before the warming treatment was applied, allowing soil and roots to acclimate to the disturbance of installation. We collected soil cores in March 2016 (Figure S1), before warming treatment began (three per plot using cores that were 5.1 cm in diameter and 10 cm in length), and we confirmed no difference in root biomass between plots assigned to the warming treatment (328 ± 32 g m^−2^) and those assigned to control (322 ± 44 g m^−2^) in the top 10 cm of soil. We began collecting root images with the minirhizotron camera in February 2017 (Figure S1) to measure root length and diameter, as described below. We also installed three root in‐growth cores (5 cm in diameter and 10 cm in length) per plot in April 2017, and these cores were harvested every 3 months until June 2018 (Figure S1) to measure root morphological traits.

### 2.3 The hurricane events

The warming treatment was paused after 11.5 months of heating on September 6th, 2017 (Figure S1), due to Hurricane Irma passing 97 km north of Puerto Rico (Category 5; mean maximum sustained wind speeds of 254 km h^−1^ or higher) and Hurricane María passing from southeast to northwest across the island on September 20th, 2017 (Category 4; mean maximum sustained winds of 209–251 km h^−1^). Hurricane María had an average rainfall of 500 mm in just 24 h (Pasch et al., 2019), and instantaneous tree mortality rates after these hurricanes were twice that of Hurricane Hugo in 1989 (~15%; Uriarte et al., 2019). The energy dose dissipated from the combined winds of Hurricanes Irma and María was 232 PJ m^−1^ of vertical air (Van Beusekom et al., 2018), which is equivalent to the energy released by an explosion of 55 megatons of TNT over Puerto Rico (Lugo, 2020). The research site was not accessible for root measurements between the two hurricane events because of extensive damage and safety concerns.

**FIGURE 1:**
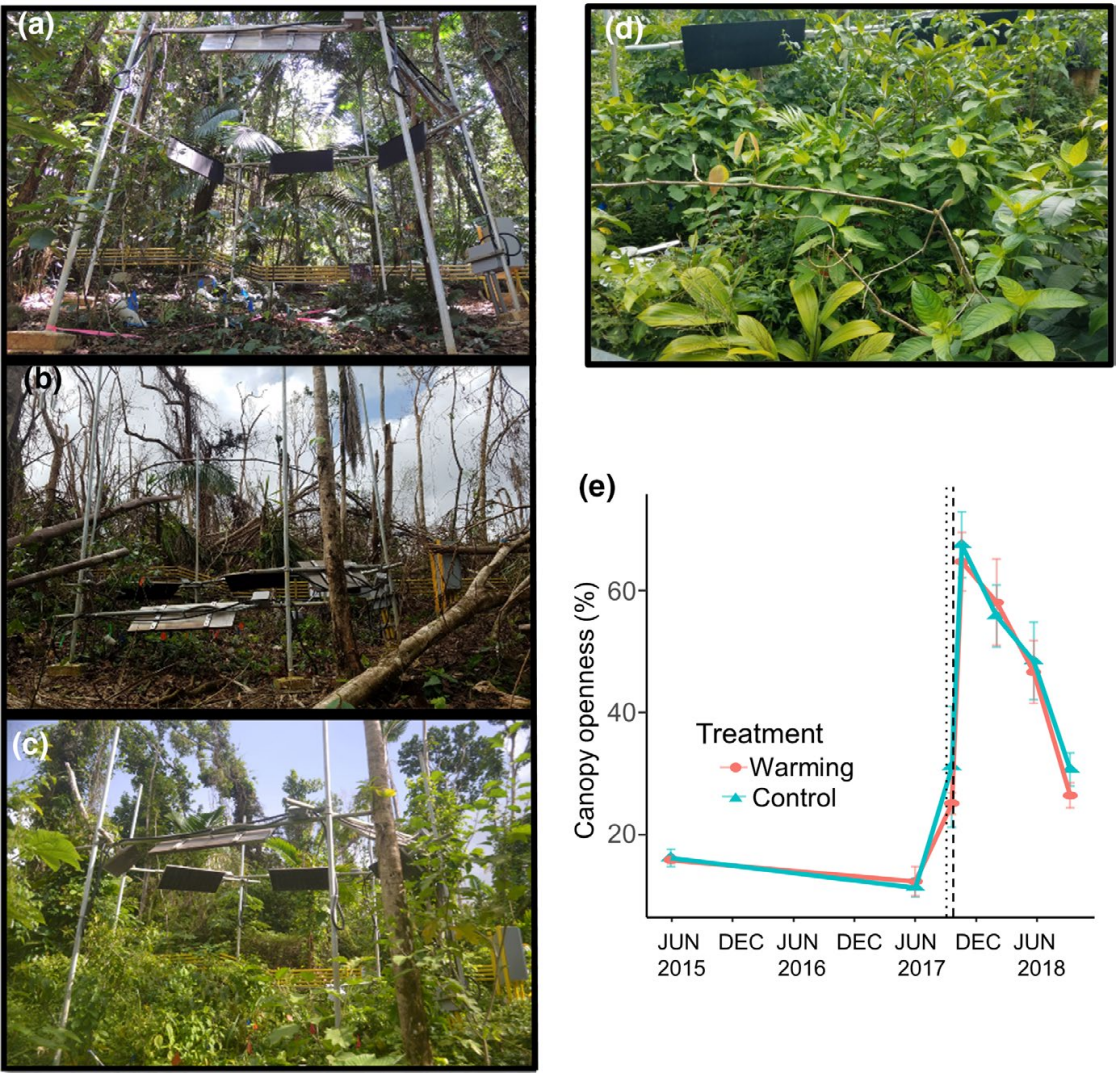
Hurricane effects on the TRACE in situ warming plots. Photograph of warming plot 2 in TRACE (a) before the hurricanes (April 2015), (b) 1 month after the hurricanes (October 2017), and (c) 9 months after the hurricanes (June 2018). (d) Photograph of the herbaceous understory canopy 5 months after the hurricanes (February 2018). (e) Canopy openness measured in warmed plots (red circles) and control plots (blue circles) in undisturbed conditions (pre‐treatment, June 2015), ~9 months after the warming treatment started (June 2017), and after the hurricanes (multiple points in 2018). Values are means and the error bars represent the standard error of plots per treatment. The dashed vertical lines indicate the timing of Hurricanes Irma and María. Adapted from Reed et al. (2020)

The hurricane events resulted in a reduction of canopy cover at our experimental site from ~90% to 30%, and there was no difference in canopy loss among the six plots (Figure 1e). Immediately following the hurricanes, the forest floor was no longer visible due to the resulting input of branches and leaf litter (Figure 1a,b). Two out of 12 minirhizotron tubes were affected by the hurricanes, one from a control plot which was broken by a tree fall, and one from a warmed plot which was slightly shifted upwards by an uprooted tree. As a result, these two tubes were removed from our analysis. The understory recovery was dominated by graminoid species during the first 4 months following the hurricanes, especially *Ichnanthus pallens* (Kennard et al., 2020). After 9 months, the understory was denser, with pioneer woody species like *Cecropia schreberiana* being more abundant (Figure 1c).

### 2.4 Root measurements

#### 2.4.1 Minirhizotron images

Images were taken every 2 weeks with a wireless manual minirhizotron camera (RhizoSystems, LLC) from continuous windows of soil area (8.3 mm wide, 6.4 mm tall) along the upper surface of the tube and for the entire tube length. There were 220 windows (images) per tube. We collected and analyzed 78,760 images in total, 36,960 of which were from the 7 months of the warming treatment, and 41,800 images from the 10 months after Hurricanes Irma and María, right before the warming treatment started again. We stopped analyzing new images at this time because we considered the re‐initiation of the warming treatment a transition to what essentially amounts to a new experiment. We analyzed images from all sessions (measurement collection day) using RootFly software version 2.0.2 (Clemson University, SC, USA, 2005–2011; https://www.quantitative‐plant.org/software/rootfly). The same person, DY, processed all images. The minirhizotron data are freely available at the NGEE‐Tropics data archive (Yaffar, 2021).

We measured length and diameter of each root segment seen on the images (all roots were included, and mycorrhizal hyphae were excluded). We used changes in root length and diameter to report root dynamics. The appearance or growth in root length and diameter was considered root production. Root disappearance, without subsequent reappearance, was considered root mortality, as in Johnson et al. (2001). We calculated root surface area using root length and diameter and applying the formula of the area of a cylinder. We calculated root biomass of each root by multiplying its length by root mass per length (RML), with the value of RML dependent on root diameter, as described by Iversen et al. (2008; see Section 2.5). The relationship between RML and diameter was determined from the soil core samples as described below. To calculate root biomass by soil surface area, we first calculated the volume of soil observed by adding a depth of field to every window. The depth of field was calibrated by comparing the root biomass of the first 10 cm of depth from the minirhizotron tubes with the root biomass of cores collected on the same dates, following the approach of Cordeiro et al. (2020). Our calibrated depth of field was 1 mm, which is close to the 0.78 mm reported by Taylor et al. (2014). Although root biomass per unit soil surface area is a standard unit reported in the literature for root standing stock, we acknowledge that the calculations used in the conversion from root morphological measurements to biomass included some general assumptions. Thus, we also report our analysis in values of root surface area (see Figure S2).

We calculated root turnover by dividing annual root production by the mean biomass (Aber et al., 1985; Aerts et al., 1992) from the 7 months before the hurricanes and 10 months after. We performed most of the analyses considering all root diameters for before and after the hurricanes. A second analysis after the hurricanes focused just on roots >2 mm diameter to distinguish woody roots from herbaceous roots (≤2 mm), following Ma et al. (2018) and visual confirmation from the in‐growth core samples. For this, we separated roots by their diameter class (<1, 1–2, >2 mm); more than 90% of roots had a diameter ≤2 mm.

#### 2.4.2 Soil cores

Soil cores were collected periodically (Figure S1) using a 5 cm diameter PVC core to 10 cm depth. We manually picked all roots from each core, separated them into coarse (>2 mm in diameter) and fine (≤2 mm in diameter), live and dead, and cleaned them gently with deionized water. We then scanned live fine roots using the WinRHIZO root‐scanning software program (Regent Instruments, Inc.) to measure total root length, average root diameter, volume, and root surface area. All roots were oven‐dried by diameter class at 65°C for at least 48 h and then weighed to obtain root biomass and calculate root biomass per volume. These data plus data from additional 30 individual roots that were collected from outside the plots (to increase sample size), that were also scanned, dried, and weighed were used to determine root mass per unit length (RML, mg cm^–1^) before and after the hurricanes, and then used to calculate root biomass of minirhizotron measurements from root length and diameter (as described above). We also used root biomass data from soil cores taken in March of 2017 for comparison with the minirhizotron root biomass data collected on the same date using only the first 10 cm of depth to confirm that general differences of treatment matched, which they did. Furthermore, measurements of soil extractable PO_4_^3−^, NH_4_^+^, NO_3_^−^, total dissolved carbon (C) and nitrogen (N), and microbial biomass N, phosphorus (P), and C concentrations from all of these cores were described by Reed et al. (2020).

#### 2.4.3 Root in‐growth cores

Root in‐growth cores were made using a rigid mesh of polypropylene and had a 5.5 cm inner diameter and 14 cm length (5 mm × 3 mm hole size). The in‐growth cores were filled with homogenized, root‐free soil that was collected adjacent to the plots. In‐growth cores were first placed in the plots in April 2017. All root in‐growth cores were collected every 3 months from installation (Figure S1), and roots were manually separated from the soil following each collection. Fine and coarse roots were divided into live and dead components. Live fine roots were scanned to measure length, diameter, and volume as described from soil cores. All roots were oven dried and weighed. After every in‐growth core was removed, we installed a new in‐growth core in the same hole. We used these data to measure morphology (root‐specific length and root diameter) before and after the hurricanes.

### 2.5 Total leaf area

To estimate total leaf area per plot, we multiplied total leaf count per species in each plot (from TRACE annual census data) by average area per leaf. Species‐specific average area per leaf values were estimated using sub‐samples of at least 10 leaves per species, collected randomly in the TRACE study area but outside the plots. Leaf area was measured using scans of leaves and Image‐J software (Rasband 1997–2018). Only woody species were included in this analysis (seedlings and saplings).

### 2.6 Statistical analyses

For all statistical analyses, we used R 3.4.4 (R Core Team and contributors worldwide). To assess changes in root morphology through time using in‐growth core data, we used a mixed‐effect model (Littel et al., 2006; Zuur et al., 2009), and tested the differences in specific root length (SRL) and fine‐root diameter over time and with the treatment effect. We included the interaction of treatment and time, and the individual effect of treatment and time, as well as a nested random term for plot (Table S1). We used the *lmer* function from the lme4 package (Bates et al., 2015).We compared the estimated marginal means of SRL and root diameter by dates using the *emmeans* function from the emmeans package. To determine the relationship between root mass per length and root diameter described by Iversen et al. (2008), we log transformed their power function and fit the resulting linear model to extract the coefficients that best described the roots collected from soil cores. These coefficients were then used in the Iversen equation to calculate root mass for each root identified in the minirhizotron. Due to differences in morphology observed before and after the hurricanes, we calculated and used different coefficients for these two periods.

We analyzed the minirhizotron data using a hierarchical mixed‐effect model for each independent variable: total root biomass, root production, and root mortality for before and after the hurricanes separately using *lme4* function from the lme4 package (Bates et al., 2015). We included only soil temperature and not air temperature due to their strong correlation. This mixed‐effect model aimed to answer if there was an effect of treatment over time on root dynamics; thus, it included the following independent variables: soil temperature and moisture, and the interaction between treatment and session. Soil moisture and temperature were taken as the average value for the period of time since the previous session. We used a nested random term for plot. We checked collinearity in each model using the *check.collinearity* function (Lüdecke et al., 2020). Temperature presented high collinearity with session and treatment before the hurricanes, and it was removed from the pre‐hurricane models. We combined all data from each tube and averaged by plot since soil moisture and soil temperature data were plot‐level variables. We also averaged soil temperature and moisture across different depths for this analysis. We employed a top‐down strategy for model selection, and we built two‐level mixed models. We considered the model with the lowest BIC as the best fit model. All models with a ∆BIC value <2 were considered to have equivalent levels of support (Burnham & Anderson, 2004); otherwise, we considered the simplest model to be the best fit. Non‐normal data were log‐transformed before analysis. We used QQ plots and Shapiro–Wilk test to evaluate all the assumptions for normality. Additionally, we ran these models on only coarse root biomass for after the hurricanes to assess the potential influence of post‐hurricane plant community (herbaceous vs. woody) on our dependent variables (Ma et al., 2018). Statistical models are presented in full in Table S2.

We performed multiple linear models using the *lm* function from the stats package (Chambers, 2017) to test the relationship of soil parameters with total root production and mortality during the warming treatment pre‐hurricanes (September of 2017) and after 10 months post‐hurricanes (June of 2018). Soil parameters included soil extractable and microbial nutrient concentrations (NH_4_^+^, NO_3_^−^, PO_4_^3−^; microbial biomass N, P, and C) from core samples (from Reed et al., 2020), and soil temperature and moisture from the soil sensors. We also tested relationships of canopy openness (found in Reed et al., 2020), and total leaf area (Table S3) per plot with root dynamics using data collected in March of 2017 and March of 2018. We first tested the collinearity between the variables and removed microbial biomass C from the model, due to high collinearity with microbial biomass N and microbial biomass P. Then we ran the models with each soil and aboveground factor separately. We started with a model that included the soil nutrient, total leaf area, the treatment, and the interaction of the factor with treatment. However, since we found no significant effect of the interaction, we dropped it. We also dropped treatment for those models where treatment was not significant (Table S4).

We modeled root vertical distribution using the Gale and Grigal (1987) asymptotic equation described in Jackson et al. (1997), in which low beta values represent shallower rooting. We used cumulative fraction of biomass from our minirhizotron data by treatment and before and after the hurricanes. The raw data and R code supporting this study are freely available on the DOE‐supported NGEE‐Tropics data archive (Yaffar, 2021).

## 3 RESULTS

### 3.1 Root dynamics response to warming treatment

**FIGURE 2:**
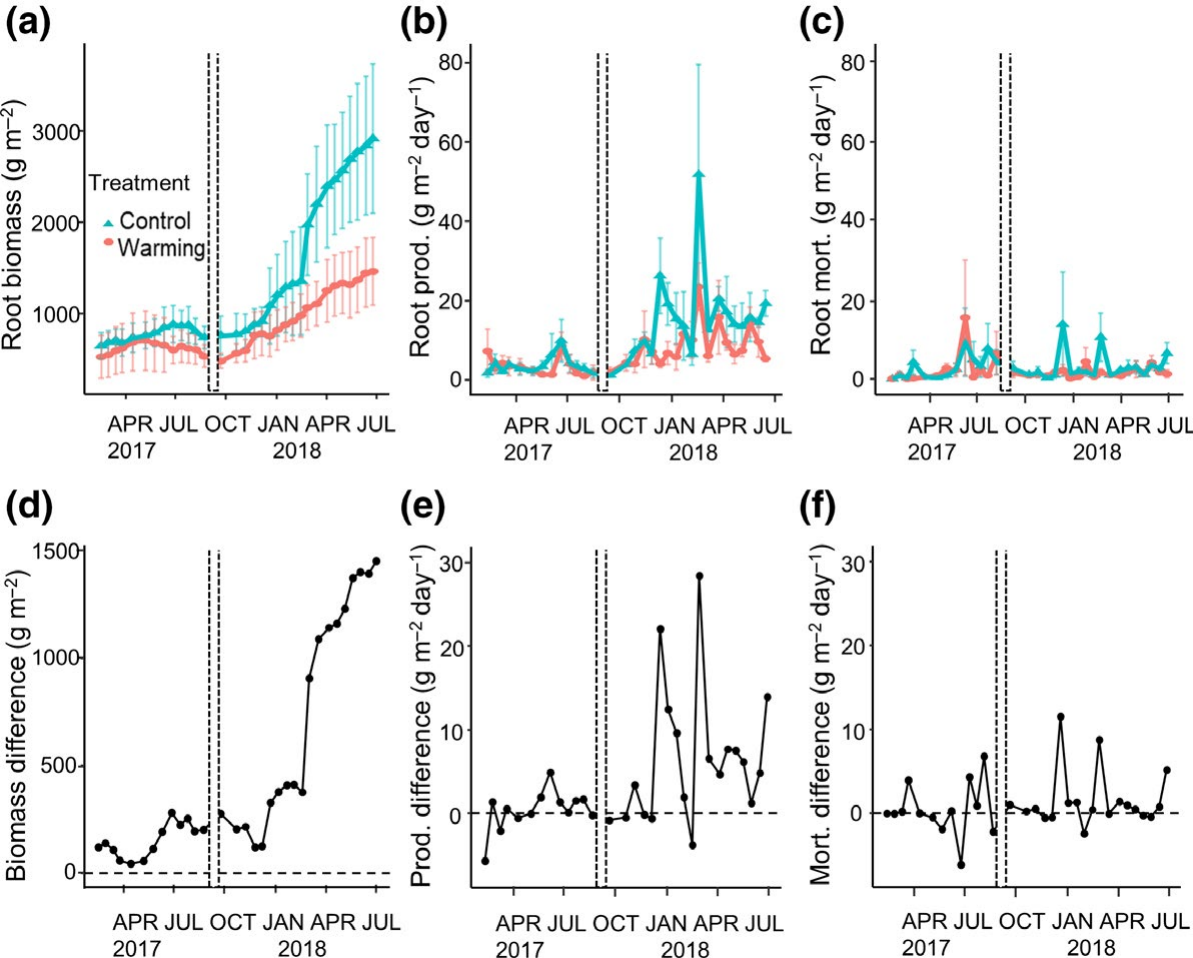
Root dynamics in response to warming and hurricane disturbance. The *x*‐axes represent the midpoint between one collection date and the following collection date. The top panels represent (a) root biomass, (b) root production, and (c) root mortality through time, as measured using the minirhizotron method. The dashed vertical lines indicate when Hurricane Irma and María struck Puerto Rico. All values are means ± standard error. The bottom panels represent the difference between control and warmed plots for (d) mean root biomass, (e) mean root production, and (f) mean root mortality through time. Red circles represent warmed plots and blue triangles represent control plots

Root biomass in control plots increased during the 7 months of the warming experiment prior to the hurricanes. In contrast, root biomass in the warmed plots declined over the same 7‐month period (Figure 2a). The initial root biomass (February 2017) was 649 ± 143 g m^−2^ in the control plots and 526 ± 237 g m^−2^ in the warmed plots; final values in the last full session before the hurricanes passed over the island (September 2017) were 739 ± 213 g m^−2^ in control plots and 535 ± 81 g m^−2^ in the warmed plots. Through the mixed‐effect model, we were able to test the difference in root biomass in control plots compared to warming plots over time (Figure 2d; treatment × time, *p* < .06), and it was primarily a reflection of the difference in productivity (Figure 2e, time × treatment, *p* < .08, Table S2) rather than mortality (Figure 2f). Production (Figure 2b) and mortality (Figure 2c) were highly variable through time (CV of 63%, and 130%, respectively). There was no significant time × treatment interaction on root mortality (Table S2). The best fit model from the mixed‐effects model analysis included treatment (warming), time (session), and their interaction (Table S2); soil moisture was not included in the best fit model and temperature was removed due to collinearity. Root turnover was 1.97 year^−1^ for the 7 months of the warming experiment (1.93 year^−1^ for control plots and 2.07 year^−1^ for warmed plots). Analyses by root surface area presented a very similar pattern to root biomass (Figure S2).

**FIGURE 3:**
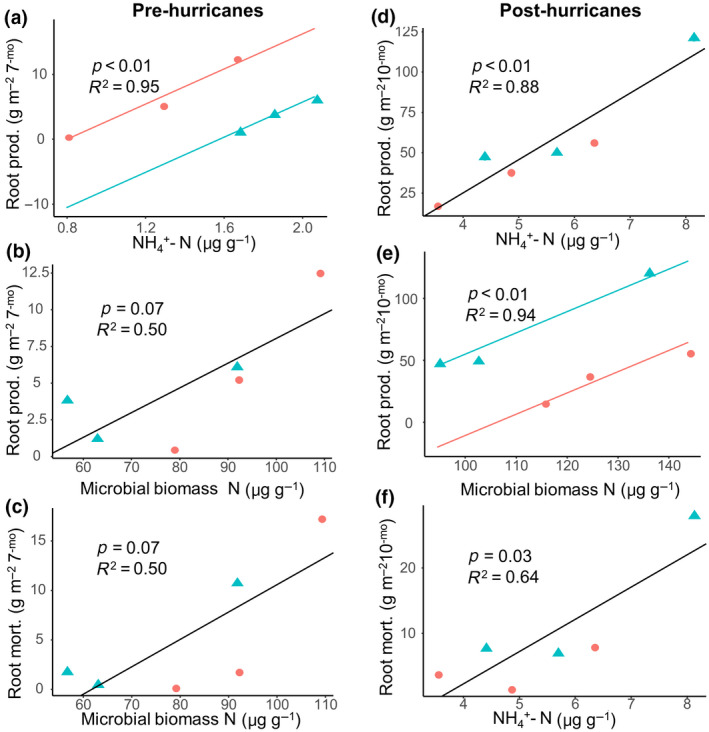
Environmental factors related to root dynamics. Significant regressions between root production and mortality during the 7 months before and 10 months after the hurricanes with soil and microbial nutrient concentrations (*n* = 6). Red circles represent warmed plots and blue triangles represent control plots, and the lines represent the linear model. Models in which treatment was insignificant are represented by a single black line, while those with a significant treatment term were represented by two lines (red and blue) representing the difference of intercepts between treatments

From the soil core data from before the hurricanes, we found that root production was positively related to soil extractable NH_4_^+^ concentration when accounting for treatment in the model (*p* < .01, *R* ^2^ = .95; Table S4; Figure 3a) across the 7 months (Table S4). We also found a positive relationship between root production and microbial biomass N concentration throughout all plots regardless of treatment (*p* = .07, *R* ^2^ = .50; Figure 3b; Table S4). Microbial biomass N concentration was also positively related to root mortality throughout all plots when not considering treatment in the model (*p* = .07, *R* ^2^ = .50; Figure 3c; Table S4). No aboveground variables or soil sensor variables showed a relationship with root production before the hurricanes.

### 3.2 Root dynamics response to the hurricanes and the warming treatment legacy

During the 10 months following hurricanes Irma and María, root biomass increased 2.8‐fold in control plots from 774 ± 223 g m^−2^ in the first full session after the hurricanes to 2918 ± 819 g m^−2^ in the last session and 1.6‐fold in warmed plots from 569 ± 102 to 1465 ± 369 g m^−2^ (Figure 2a). This increase over time was greater for the control plots than the warmed plots (time × treatment, *p* < .01, Table S2). Soil moisture and root biomass were negatively related (*p* = .01; Table S2). The largest difference in root biomass between control and warmed plots was attributable to increased production in controls from 4 + 0.4 g m^−2^ day^−1^ right after the hurricanes (October 2017) to 19.3 ± 3.2 g m^−2^ day^−1^ 10 months after the hurricanes (June 2018, Figure 2b,e) compared to 4.3 ± 2 g m^−2^ day^−1^ and 5.3 ± 0.6 g m^−2^ day^−1^, respectively, in previously warmed plots. However, there was a large variation in production by time (session) after the hurricanes (Figure 2b,e). Soil temperature was included in the best fit model and influenced root production; there was greater production at lower temperatures (*p* < .01; Table S2). Root mortality also increased with time (*p* < .01) yet none of the other variables affected root mortality (Table S2). Treatment affected coarse root biomass over time as well (*p* < .01; Table S2). Root turnover was 3.6 year^−1^ for the 10 months after the hurricanes (3.59 year^−1^ for control plots and 3.65 year^−1^ for previously warmed plots).

Root production after the hurricanes was positively related to soil extractable NH_4_^+^ concentration throughout all the plots and when not considering treatment in the mixed‐effect model (*p* < .01, *R* ^2^ = .88; Figure 3d; Table S4). Microbial biomass N concentration was positively related to root production only when treatment was considered in the model (*p* < .01, *R* ^2^ = .94; Figure 3e; Table S4). Soil extractable NH_4_^+^ concentration was positively related to root mortality throughout all plots and when not considering treatment in the model (*p* = .03, *R* ^2^ = .64; Figure 3f; Table S4). No aboveground variables showed a correlation with root production after the hurricanes.

### 3.3 Root depth distribution

**FIGURE 4:**
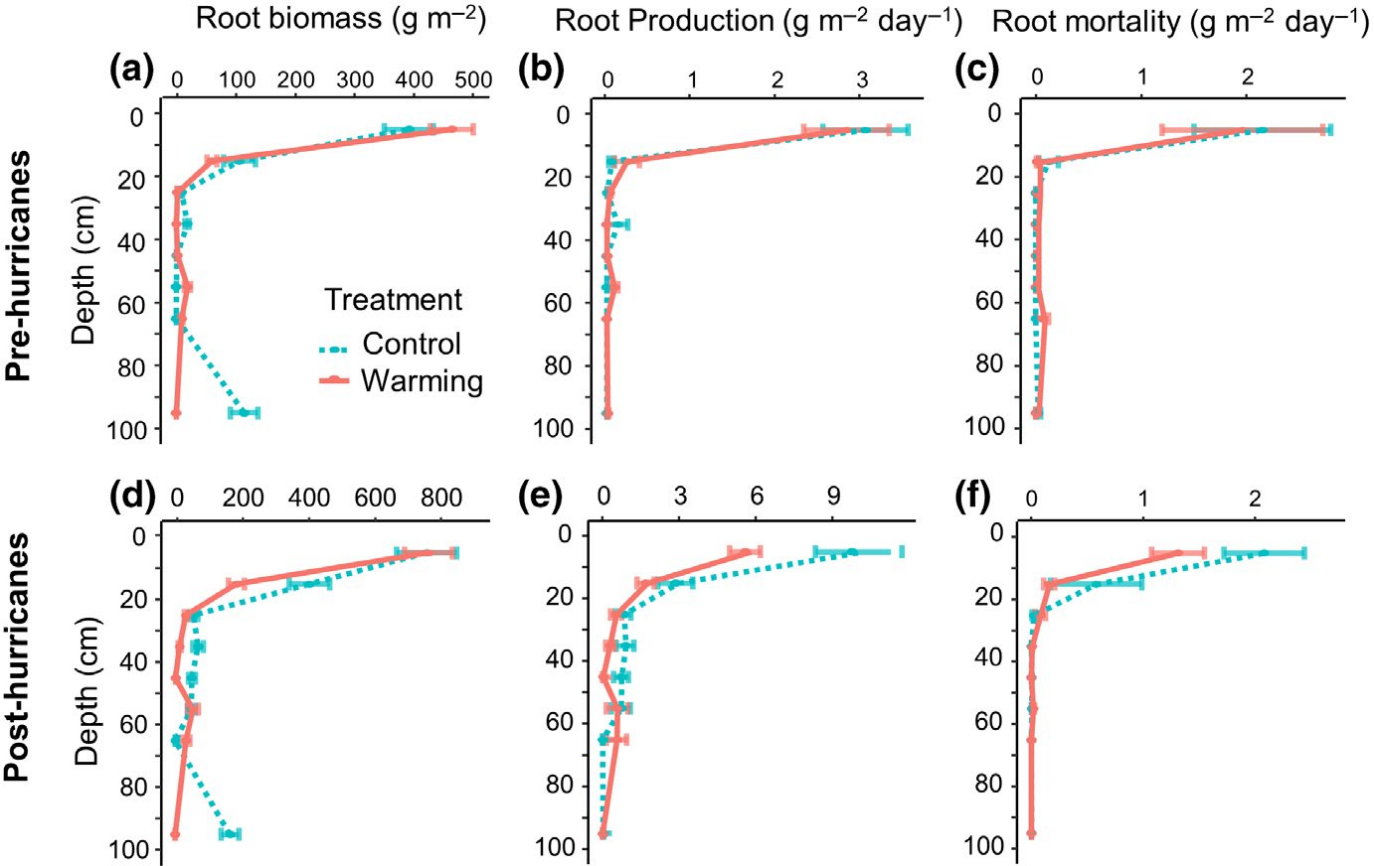
Depth distribution of roots before and after hurricanes. Root biomass, root production, and root mortality before the hurricanes (a–c) and after the hurricanes (d–f) by depth, as measured by the minirhizotron method. Circles represent the mean and the error bars are standard error. Red circles with solid lines depict data from the warmed plots and blue circles with dashed lines depict control plot data

Root biomass was predominantly in the top 30 cm of soil (Figure S3). The cumulative root biomass distribution beta coefficient was on average .92. Based on the beta coefficient, control plots seemed to have a deeper root distribution (beta = .95 before hurricanes and beta = .96 after hurricanes; Figure S3) compared to warmed plots (beta = .84 before hurricanes and beta = .90 after hurricanes; Figure S3), but this was mainly influenced by existent thicker roots in only one control plot (Figure 4). Root production was detected as deep as 90 cm, and root mortality was observed at 100 cm (Figure 4). Overall, patterns of root depth distribution did not differ with treatment before the hurricanes (Figure 4a–c). Root biomass increased two‐fold after the hurricanes in the top 10 cm of soil. However, the greatest increase was in the next two depth ranges (10–20 and 20–30 cm), where root biomass was five‐fold greater after the hurricane than before (Figure 4d). Root production increased after the hurricanes, especially for the control plots where production was two‐fold in the first 10 cm of depth, and more than 50 times that of the next 20 cm of depth (10–30 cm), compared to values before the hurricanes (Figure 4e). Root mortality also increased after the hurricanes, with mortality rates ranging from 4 to 32 times higher compared to those observed before the hurricanes, although not in the first 10 cm of depth (Figure 4f).

### 3.4 Root morphology changes with hurricane disturbance from soil in‐growth cores

**FIGURE 5:**
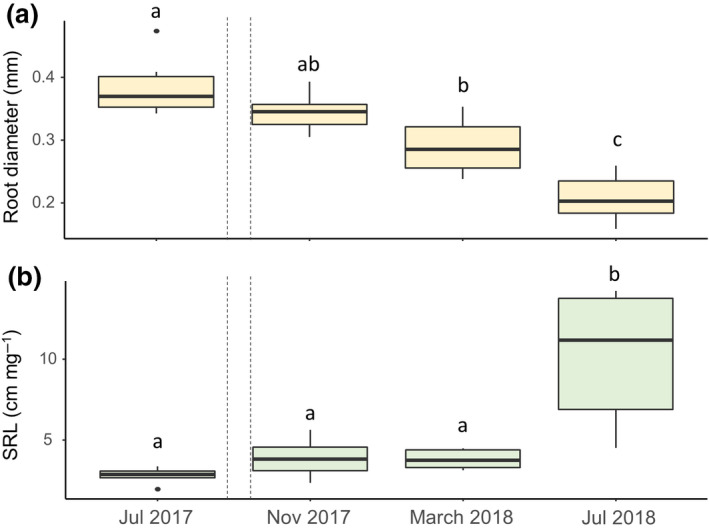
Fine‐root morphology changes with hurricane disturbance. Morphology of new fine roots extracted from in‐growth cores every 3 months before and after the hurricanes (dashed lines represent Hurricane Irma and María). (a) Mean fine‐root diameter decreased (*p* < .05) following the hurricanes as assessed using new roots collected from the in‐growth cores. (b) Mean specific root length increased (*p* < .05) following the hurricanes. Different letters represent significant differences in estimated marginal means by date

Fine‐root morphological traits measured in in‐growth cores changed over time. Fine‐root diameter decreased significantly after the hurricanes (*p* < .01; Figure 5a; Table S1), and this change was not related to the warming treatment. Specific root length (SRL) was significantly greater 10 months after the hurricanes compared to before the hurricane disturbance events (*p* < .01; Figure 5b; Table S1) and was again not related to the warming treatment (Table S1).

## 4 DISCUSSION

Understanding forest responses to multiple disturbances is not straightforward, especially in tropical ecosystems, where more carbon is cycled than in any other biome and where field‐based experimental studies are less common relative to higher latitudes (Cavaleri et al., 2015; Ratajczak et al., 2018; Saugier et al., 2001; Wood et al., 2019). Although changes to the aboveground structure of a tropical forest following a major tropical hurricane and sometimes in response to warming can be seen with the naked eye, the multifaceted response of the rhizosphere is not easily perceived. Our study measured root dynamics in response to understory and soil warming (+4°C), the effects of two consecutive hurricane events, the legacy effects of the prior warming on root recovery, and the environmental factors that may help predict these responses. We found that, prior to the hurricanes, root biomass and root production were negatively affected by the warming treatment through time. Following the hurricanes, root biomass increased across both treatments; yet, the increase was much less in previously warmed plots than controls, despite similar climatic conditions without warming treatment for the year of hurricane recovery. These patterns suggest that a warming legacy negatively affected root biomass recovery following hurricane disturbances. Furthermore, had the warming treatment continued after the hurricanes, the direct effect of warming might have been expected to compound this negative legacy effect. We also recognize that our results cannot necessarily be assumed to represent the integrated responses of a forest in the warmer climate of the future. As with other forest warming studies (e.g., Nottingham et al., 2020), we were not able to warm the entire forest, and the root responses we observed might be different if, for example, carbon allocation to roots was altered by warming of the forest canopy.

### 4.1 Responses of root dynamics to the warming treatment

The values we obtained for root biomass overall and root production pre‐hurricanes were within the ranges of values reported in other tropical studies (Berish, 1982; Cuevas et al., 1991; Jackson et al., 1997; Yaffar & Norby, 2020). Rooting depth was similar to what other studies found previously in Puerto Rico (30 cm; Frangi & Lugo, 1985; Yaffar & Norby, 2020). Root distribution patterns were similar in warmed and control plots, possibly because there was no secondary drought effect of the warming (Kimball et al., 2018; Lynch & Wojciechowski, 2015; Wood et al., 2019).

We found significant effects of warming on root production and root biomass throughout the 7‐month measurement period prior to the hurricane disturbance. We found that root production overall was positively related to soil extractable NH_4_^+^ and microbial biomass N concentrations. According to Reed et al. (2020), control plots had greater extractable NH_4_^+^ than warmed plots before the hurricanes, which could have influenced the root production differences with treatment. Previous studies in temperate and boreal forests have found a positive response of root production to warming as a result of increased nutrient supply (Majdi & Öhrvik, 2004; Pugnaire et al., 2019; Wan et al., 2004; Zhou et al., 2012). Alternatively, the soil extractable NH_4_^+^ concentration could have been a response to the increase of root production, but we cannot confirm the direction of the relationship from our study.

Temperature has myriad effects on many ecosystem processes; the negative effect of the warming treatment on root dynamics could have resulted from direct effects of increased temperature on physiological function (e.g., affecting carbon supply to roots or metabolic processes in roots) or from indirect effects on the soil chemical, biological, or physical environment (e.g., altered nutrient availability, microbial activity, or soil water content) (Reed et al., 2020). Other experiments have reported increased root production in response to imposed warming (Fitter et al., 1999; Forbes et al., 1997; Malhotra et al., 2020; Wan et al., 2004; Wang et al., 2021), but they were all located in temperate or boreal biomes (Cavaleri et al., 2015; Rustad et al., 2001) where temperatures reach below freezing and precipitation is usually no greater than 9.5 mm day^−1^ (Whitaker, 1975). Air temperatures at our study site in Puerto Rico fluctuate between 23°C (February) and 27°C (October), and rainfall is generally not limiting plant growth (annual average 8–12 mm day^−1^; Harris et al., 2012); hence, the temperature effects on plant‐soil feedbacks are distinct from higher latitude ecosystems (Balser & Wixon, 2009).

The climate regime of our site in the tropics might have played an important role in the root dynamics response to warming compared to other warming experiments. It is known that the uppermost canopy layer in our site was already exceeding its photosynthetic temperature optima (*T*_opt_; Mau et al., 2018; Miller et al., 2021). Although the understory is usually under its *T*_opt_ (<30°C; Carter et al., 2020; Miller et al., 2021), during some hours of summer days surface temperature exceeded this *T*_opt_ in the warmed plots (3% of the summer period). However, surface temperature of control plots did not exceed 27°C. This difference in surface temperature might have had an effect on photosynthesis rates of the understory, potentially leading to less carbon allocated to the roots and less carbon accumulated in the plants from the warmed plots (Wang et al., 2021). Carter et al. (2020) found evidence of a reduction of photosynthetic rates under the warming treatment and a lack of thermal respiratory acclimation prior to the hurricanes, which may have resulted in less carbon available for root growth.

### 4.2 Responses of root dynamics to the hurricane events

During the 10 months after Hurricanes Irma and María, root production, and root biomass increased, but biomass increased 2.8‐fold in the control plots versus 1.6‐fold in the previously warmed plots. The shift in root biomass values from before to after the hurricanes can be explained by the changes in the aboveground plant community, which we detected both in belowground measurements of morphological changes and visual qualitative cues. Grass first, and then other herbaceous plants and woody seedlings dominated the forest floor after 3–12 months from the disturbance events (Kennard et al., 2020). Roots from before the hurricanes were thicker but shorter than roots observed after the hurricanes. This change, according to an analysis of morphological differentiation of woody plant roots and herbaceous roots (Ma et al., 2018), implies a change in dominance from woody to herbaceous plants, corresponding to aboveground observations (Kennard et al., 2020). High values of root surface area after the hurricanes (45.4 m^2^ m^−2^; Figure S2) were also consistent with values obtained from tropical grassland/savanna (42.5 m^2^ m^−2^; Jackson et al., 1997). Furthermore, tree species from Puerto Rico did not show great changes in morphology after hurricane disturbances (Yaffar et al., 2021).

Considering the change in plant community, our finding that root biomass and production differed between control and previously warmed plots after the hurricanes could reflect changes in the dominance of herbaceous and graminoid plants. Thus, we separated roots into woody roots and herbaceous roots based on diameter size and visual cues. We recognize this assumption is very broad, but this approach provides us some ability to differentiate based on root “type” (Ma et al., 2018). From our in‐growth cores and soil cores, we knew that most roots belonging to herbaceous plants were thinner than 1 mm in diameter. Therefore, we assumed that roots greater than 2 mm belonged exclusively to woody plants. Since 70% of the understory plant composition after the hurricanes were from herbaceous species (Kennard et al., 2020), and due to visual observations, we infer that woody roots (>2 mm) belong to woody plants that existed before the hurricanes and thus responded to the combined effects of warming and hurricane disturbance. When running the mixed‐effects model using only roots greater than 2 mm in diameter, we still found a significant effect of treatment on root biomass through time (interaction treatment × session; Table S2) after the hurricane events. Thus, we conclude that both new herbaceous plants and woody plants responded similarly to the warming treatment legacy.

After the hurricanes, there was no significant difference in soil temperature between control plots and previously warmed plots in June 2018. There was additionally no difference in canopy openness or percent bare ground between warmed and control plots after the hurricanes. Furthermore, total leaf area per plot did not differ significantly between control and previously warmed plots either before or after the hurricanes, yet there was a trend toward twice more total leaf area in control plots compared to warmed plots. Although we expected the aboveground recovery to mirror the legacy effect of warming after the hurricanes, the measurements taken for total leaf area and canopy openness did not show strong statistical evidence of a differential effect.

The warming treatment had legacy effects on soil nutrients after the hurricanes, with greater soil extractable NO_3_^−^ and PO_4_^3−^ concentrations in plots that have been warmed and possibly greater NH_4_^+^ concentration (not significant) in control plots (Reed et al., 2020). The observed positive relationship between root production and extractable NH_4_^+^ concentrations after the hurricanes could reflect a stimulatory effect of greater nutrient availability on plant (including root) production, or conversely, a stimulation of nutrient mineralization by increased root activity. Resolution of any cause‐and‐effect relationships will require more detailed analysis of nutrient dynamics. Thus, the complexity of the biogeochemical response to hurricane disturbances versus the plant response on previously warmed plots and the direction are not completely evident, and can be happening as parallel, or as concomitant non‐additive processes.

### 4.3 Implications of multiple disturbances

The recovery of forests from disturbance is a critical aspect of forest dynamics, and it is important for societal well‐being. Hurricane disturbances in Puerto Rico are relatively frequent; current plant species are adapted accordingly to these disturbances (Miller & Lugo, 2009). The forest canopy is shaped by strong winds (Basnet et al., 1992), and the understory growth is triggered when gaps are formed, yet the responses of root systems are not as evident. It is estimated that the return interval of a hurricane the size and power of María is 86 years (Lugo, 2020). However, the intensity and frequency of these hurricanes are shown to be changing due to climate change (Intergovernmental Panel on Climate Change, 2013; Lin et al., 2016). Without sufficient time to replenish plant energy reserves, ecosystem development could be affected (Lugo, 2020). Furthermore, the interaction of natural disturbances, such as hurricanes, and climate warming can alter the ecosystem recovery and trajectory (Ratajczak et al., 2018).

Resilience and recovery of root systems are essential for the aboveground recovery after a disturbance (Uriarte et al., 2019; Vargas et al., 2010). Root system responses can also affect the residence time of carbon in the soils (Kell, 2012) and soil stability (Gyssels et al., 2005), with additional implications for the forest community, Carbon cycling, and human society. Our study showed that understory and soil warming can negatively affect the belowground plant component of small patches in a tropical forest, and the effects of warming can further slow the trajectory of belowground recovery from hurricane disturbance. Inferring causation will help us anticipate future and multiple disturbances. The study presented here provides valuable empirical data that can be used to model future scenarios, help elucidate the key environmental variables interacting on the belowground ecosystem, and provide a baseline for future larger warming plots in the tropics with adult trees.

## CONFLICT OF INTEREST

The authors declare that they have no conflict of interest.

## AUTHORS’ CONTRIBUTION

DY participated in the field equipment installation, took the field and laboratory measurements, analyzed the data, and wrote the paper. TEW, SCR, and MAC developed the concept for the project, installed field equipment, provided environmental data, reviewed, and edited the paper. BLB assisted in data analysis, reviewed, and edited the paper. RJN installed the field equipment, helped developed the concept for the project, reviewed, and edited the paper.

## Supporting information

Supplementary Material

## Data Availability

The raw data supporting this study are freely available on the DOE‐supported NGEE‐Tropics data archive (https://doi.org/10.15486/ngt/1582598).
