# Supplementary material for: Experimental warming and its legacy effects on root dynamics following two hurricane disturbances in a wet tropical forest
Source: Glob Chang Biol. 2021 Sep 26;27(24):6423–35. doi: 10.1111/gcb.15870 (PMC9293463; doi:10.1111/gcb.15870)

**SUPPLEMENTS**

Table S1. Mixed effect models for specific root length (SRL) and root diameter; model; dependent variables; fixed effects; random effects; P values.

| **Root trait** | **Model (lmer)** | **Dependent variable** | **Fixed effects** | **Random effect** | **P value** |
| --- | --- | --- | --- | --- | --- |
|  |  |  |  |  |  |
| SRL | SRL~Time+ treatment+ time:treatment+  (1\|Plot) | SRL | Time | Plot/treatment | <0.01 |
|  |  |  | Treatment  Time:treatment |  | 0.18  0.37 |
| Root diameter | Root diameter~Time+ treatment+ time:treatment+ (1\|Plot) | Root diameter | Time | Plot/treatment | <0.01 |
|  |  |  | Treatment  Time:treatment |  | 0.97  0.71 |

Table S2: Mixed effect models; dependent variables; fixed effects; random effects; P values; R^2^ marginal and R^2^ conditional; coefficient estimates

| **Time** | **Model (lmer)** | **Dependent variable** | **Fixed effects** | **Random effect** | **P value** | **R^2^m/R^2^c** | **Estimate** |
| --- | --- | --- | --- | --- | --- | --- | --- |
|  |  |  |  |  |  |  |  |
| Before | Root biomass~treatment + session + (1\|plot) | Root biomass | Treatment | Plot | 0.57 | 0.05/  0.89 | 8.32 |
|  |  |  | Session |  | 0.02 |  | -154.7 |
| Before | Root production~treatment + session + (1\|plot) | Root production | Treatment | Plot | 0.7 | 0.07/ 0.5 | -0.6 |
|  |  |  | Session |  | 0.002 |  | -0.17 |
| Before | Root mortality~treatment + session + (1\|plot) | Root mortality | Treatment | Plot | 0.9 | 0.16/ 0.5 | 0.12 |
|  |  |  | Session |  | <0.001 |  | 0.46 |
| Before | Root biomass~treatment+ session+ treatment:session + (1\|plot) | Root biomass | Treatment | Plot | 0.57 | 0.06/ 0.9 | -46.7 |
|  |  |  | Session |  | 0.02 |  | 15 |
|  |  |  | Treatment:session |  | 0.058 |  | -13.5 |
| Before | Root production~ treatment+ session+ treatment:session+ (1\|plot) | Root production | Treatment | Plot | 0.7 | 0.08/ 0.5 | 0.9 |
|  |  |  | Session |  | 0.002 |  | -0.07 |
|  |  |  | Treatment:session |  | 0.08 |  | -0.19 |
| Before | Root mortality~ treatment+ session+ treatment:session+ (1\|plot) | Root mortality | Treatment | Plot | 0.9 | 0.16/ 0.5 | 0.36 |
|  |  |  | Session |  | <0.001 |  | 0.48 |
|  |  |  | Treatment:session |  | 0.86 |  | -0.03 |
| After | Root biomass~treatment + session +moisture + (1\|plot) | Root biomass | Treatment | Plot | 0.1 | 0.5/ 0.8 | -702.1 |
|  |  |  | Session |  | <0.001 |  | 83.9 |
|  |  |  | Moisture |  | 0.01 |  | -0.95 |
| After | Coarse root biomass~treatment+ session+ (1\|plot) | Coarse root biomass | Treatment | Plot | 0.18 | 0.2/ 0.7 | -659.1 |
|  |  |  | Session |  | <0.001 |  | 37.9 |
| After | Root production~treatment + session+ temperature + (1\|plot) | Root production | Treatment | Plot | 0.6 | 0.25/ 0.27 | -0.1 |
|  |  |  | Session |  | <0.001 |  | 0.08 |
|  |  |  | Temperature |  | <0.001 |  | -0.3 |
| After | Root mortality~treatment + session + (1\|plot) | Root mortality | Treatment | Plot | 0.1 | 0.15/ 0.3 | -1.24 |
|  |  |  | Session |  | <0.01 |  | 0.14 |
| After | Root biomass~treatment+ session+ treatment:session + (1\|plot) | Root biomass | Treatment | Plot | 0.1 | 0.5/ 0.8 | 126 |
|  |  |  | Session |  | <0.001 |  | 142 |
|  |  |  | Treatment:session |  | <0.001 |  | -86.78 |
| After | Coarse root biomass~treatment+ session+ treatment:session + (1\|plot) | Coarse root biomass | Treatment | Plot | 0.2 | 0.3/ 0.8 | 825.3 |
|  |  |  | Session |  | <0.001 |  | 68.8 |
|  |  |  | Treatment:session |  | <0.001 |  | -61.8 |
| After | Root production~ treatment+ session+ treatment:session+ temperature +(1\|plot) | Root production | Treatment | Plot | 0.6 | 0.25/ 0.27 | -0.2 |
|  |  |  | Session |  | <0.001 |  | 0.07 |
|  |  |  | Treatment:session |  | 0.7 |  | 0.01 |
|  |  |  | Temperature |  | <0.001 |  | -0.31 |
| After | Root mortality~ treatment+ session+ treatment:session+ (1\|plot) | Root mortality | Treatment | Plot | 0.1 | 0.15/ 0.3 | -1.93 |
|  |  |  | Session |  | <0.001 |  | 0.1 |
|  |  |  | Treatment:session |  | 0.3 |  | 0.07 |

Table S3: Total leaf area (m^2^ m^-2^) and treatment since conditions of pre-warming (2016), through warming (2017), post-hurricane but unwarmed (2018), up to the re-start of the warming treatment after the hurricanes (2019)

| **Conditions** | **year** | **Plot ID** | **Assigned treatment** | **Total leaf area (m^2^ m^-2^)** |
| --- | --- | --- | --- | --- |
| Pre-warming,  pre-hurricane |  | 1 | Control | 0.29 |
|  |  | 2 | Unwarmed | 0.17 |
|  | 2016 | 3 | Control | 0.31 |
|  |  | 4 | Unwarmed | 0.24 |
|  |  | 5 | Control | 0.43 |
|  |  | 6 | Unwarmed | 0.14 |
| Warming,  pre-hurricane |  | 1 | Control | 0.39 |
|  |  | 2 | Warmed | 0.26 |
|  | 2017 | 3 | Control | 0.50 |
|  |  | 4 | Warmed | 0.41 |
|  |  | 5 | Control | 0.77 |
|  |  | 6 | Warmed | 0.11 |
| Post-warming, post-hurricane |  | 1 | Control | 0.33 |
|  |  | 2 | Unwarmed | 0.51 |
|  | 2018 | 3 | Control | 0.93 |
|  |  | 4 | Unwarmed | 0.36 |
|  |  | 5 | Control | 0.98 |
|  |  | 6 | Unwarmed | 0.42 |
| Warming,  post-hurricane |  | 1 | Control | 0.43 |
|  |  | 2 | Warmed | 0.37 |
|  | 2019 | 3 | Control | 0.52 |
|  |  | 4 | Warmed | 0.32 |
|  |  | 5 | Control | 0.84 |
|  |  | 6 | Warmed | 0.63 |

Table S4. Linear models for before and after the hurricane events. Each model has a sample size of 6. Model; dependent variables; independent variables; P values; R^2^; coefficient estimates

| **Time from hurricanes** | **Model (lm)** | **Dependent variable** | **Independent variable** | **P value** | **R^2^** | **Estimate** |
| --- | --- | --- | --- | --- | --- | --- |
| Before | Root production~ NH4+ treatment | Root production | NH4 | <0.01 | 0.95 | 13.49 |
|  |  |  | Treatment | <0.01 |  | -10.52 |
| Before | Root production~ microbial N | Root production | Microbial N | 0.07 | 0.50 | 0.17 |
| Before | Root mortality~ microbial N | Root mortality | Microbial N | 0.07 | 0.50 | 0.28 |
| After | Root production~ NH4 | Root production | NH4 | 0.02 | 0.88 | 18.58 |
| After | Root production~ microbial N+ treatment | Root production | Microbial N | <0.01 | 0.94 | 1.72 |
| After | Root mortality~ NH4 | Root mortality | Treatment  NH4 | <0.01  0.03 | 0.64 | 65.64  4.93 |

Figure S1. Timeline of the events and measurements that took place in this study during the pre-warming treatment, the warming treatment, and the post-warming treatment. Open boxes represent events that took place on a specific date, while filled boxes represent those measurements that took place during a period delimited by the box itself.


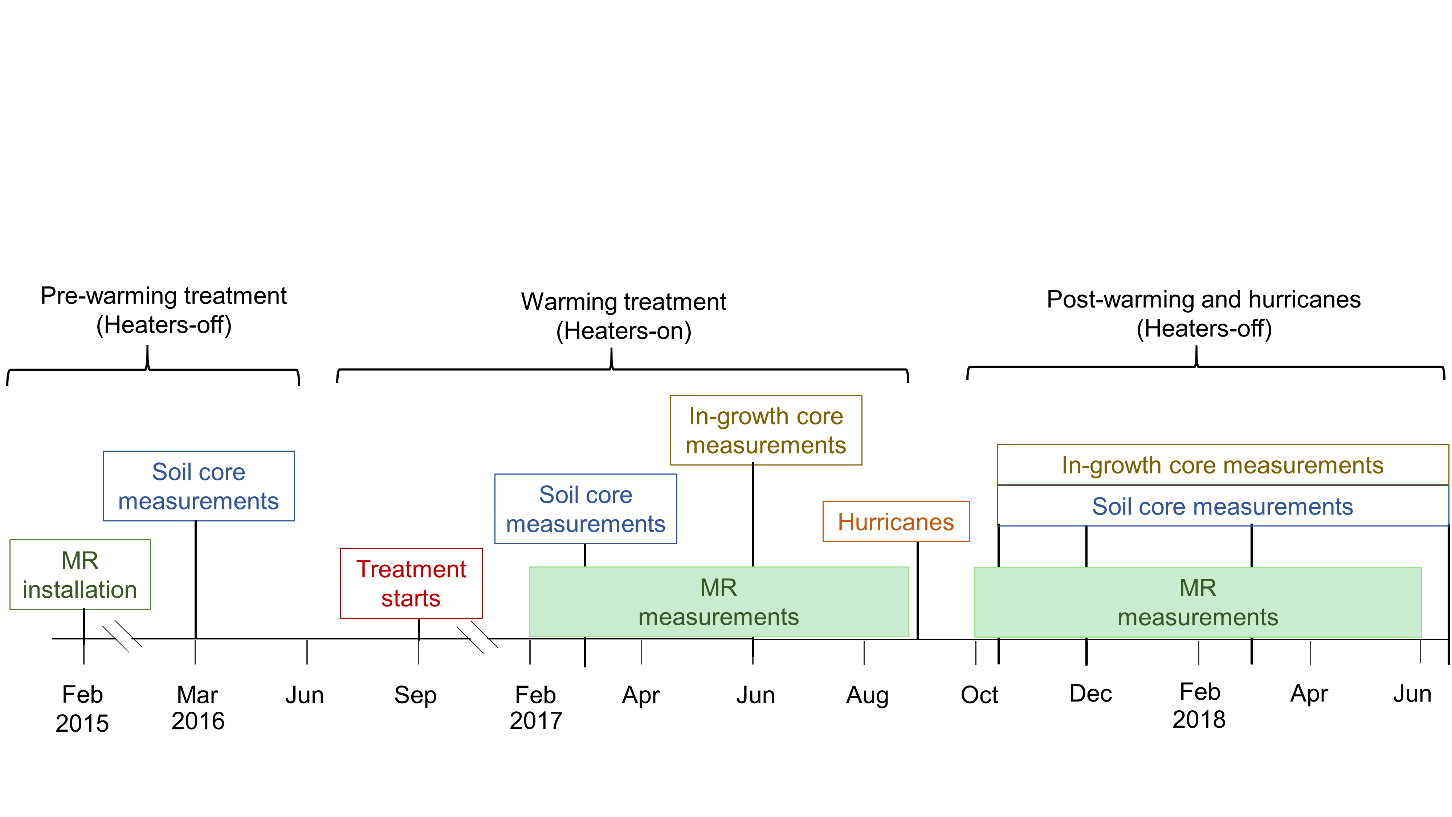


Figure S2: Average root surface area (m^2^ m^-2^) per treatment (red circles are warming plots and blue triangles are control plots) and in time. In the x axis are the collection dates (mm-yy). The dashed line represents the date when Hurricane María hit Puerto Rico.


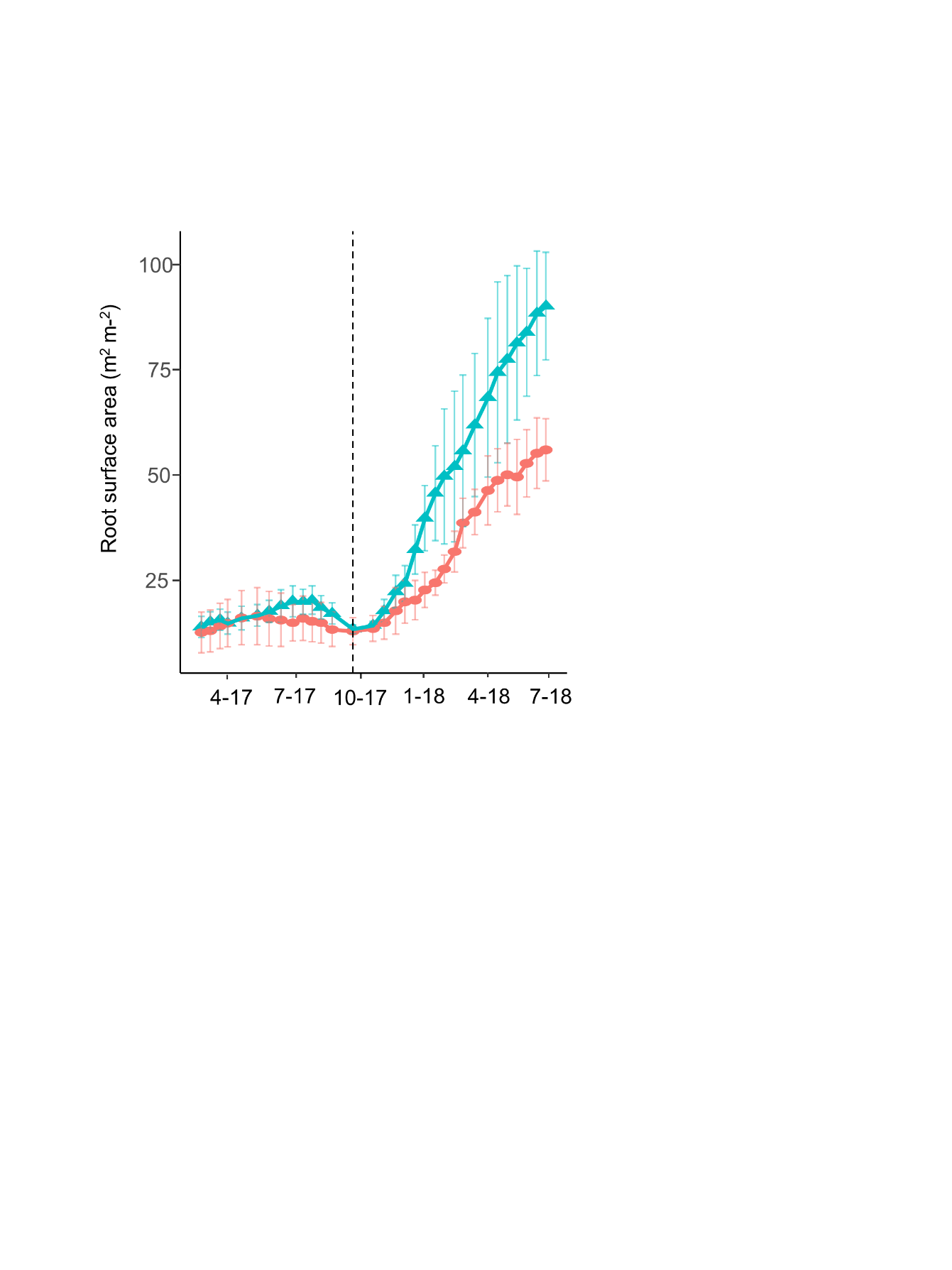


Figure S3: Root vertical distribution by treatment and before and after the hurricanes, as measured by the minirhizotron method. Control plots are shown in blue, warmed plots are shown in red. Before the hurricanes data are represented by a triangle, and after data are represented by a circle.


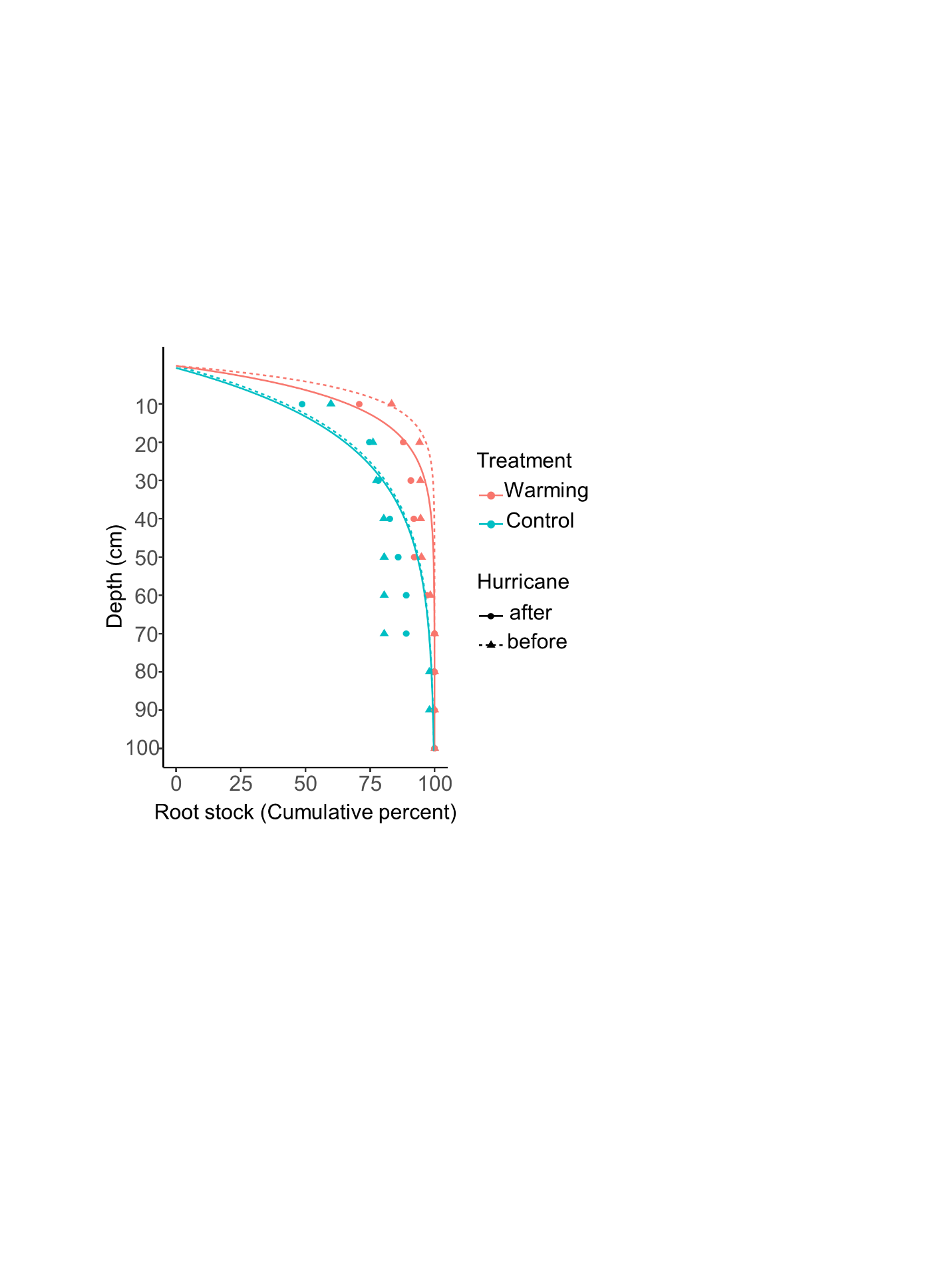

Supplement: Supplementary file 1 — Supplementary Material [file GCB-27-6423-s001.docx]
